# Graphene/PbS quantum dot hybrid structure for application in near-infrared photodetectors

**DOI:** 10.1038/s41598-020-69302-6

**Published:** 2020-07-27

**Authors:** Hyun Jeong, Jung Hoon Song, Sohee Jeong, Won Seok Chang

**Affiliations:** 1 Department of Nano Manufacturing Technology, Korea Institute of Machinery and Materials (KIMM), Daejeon, 34103 Republic of Korea; 2 Department of Energy Science (DOES), Sungkyunkwan University (SKKU), Suwon, 16419 Republic of Korea; 3 Department of Nano-Mechatronics, Korea University of Science and Technology (UST), Daejeon, 34113 Republic of Korea

**Keywords:** Quantum dots, Electronic properties and devices, Optical sensors

## Abstract

A graphene-PbS quantum dot (QD) composite for application in high-performance near-infrared (NIR) photodetectors (PDs) is proposed in this study. A single-layer graphene flake and oleic acid-capped PbS QD composite is fabricated through the conventional sonication process, in hexane solution. Field emission scanning electron microscopy images of the graphene-PbS QD composite dispersed on a glass substrate confirm that the composite contains both aggregated graphene flakes and single-layer graphene with wrinkles; Transmission electron microscopy images reveal close packing with uniform size. The increased absorbance and quenched photoluminescence intensity of the graphene-PbS QD composite supports enhanced photoinduced charge transfer between graphene and the PbS QDs. Moreover, the specific Raman mode of the PbS QDs, embedded in the spectrum, is enhanced by combination with graphene, which can be interpreted by SERS as relevant to the photoinduced charge transfer between the Pbs QDs and graphene. For device application, a PD structure comprised by graphene-PbS QDs is fabricated. The photocurrent of the PD is measured using a conventional probe station with a 980-nm NIR laser diode. In the fabricated PD comprising graphene-PbS QDs, five-times higher photocurrent, 22% faster rise time, and 47% faster decay time are observed, compared to that comprising PbS QDs alone. This establishes the potential of the graphene-PbS QD composite for application in ultrathin, flexible, high-performance NIR PDs.

## Introduction

Low-dimensional materials are suitable for application in flexible devices because of their atomic-scale dimensions^1–3^. In particular, two-dimensional layered materials and zero-dimensional quantum dots have attracted significant attention for optoelectronic applications because of their excellent optical and electrical properties^4–11^. Graphene is a representative metallic layered material consisting of carbon atoms arranged in a hexagonal lattice with strong inplane chemical bonds^12–14^. Although graphene has high electron concentration, weak light absorption and fast carrier recombination limit its application in optoelectronics^15,16^. PbS colloidal quantum dots (QDs) are promising semiconductors for application as active material in near-infrared optoelectronics because of their small and tunable direct band gap^17,18^. Moreover, PbS QDs have outstanding optical and electrical properties due to which their application can be expanded to photodetectors (PDs), light emitting devices, field effect transistors, and photovoltaics^19–22^. The different work function and carrier concentration of graphene and the PbS QD enable photoinduced charge transfer when these two materials are in physical contact^23–25^. For application in photoresponsive devices, this phenomenon can enhance the photoresponsivity and improve the response time of the PD.

One of primary mechanisms of surface-enhanced Raman scattering (SERS) is chemical enhancement that involves photoinduced charge transfer between inhomogeneous substances^26^. Photoinduced charge transfer can be the dominant contribution to the surface-enhanced Raman signal on the semiconductor substrate^27^. Therefore, the study of the SERS of graphene and PbS QDs is significant for the analysis of the photoinduced charge transfer and Raman spectroscopy.

The hybrid structure comprising graphene and PbS QDs is atomically thin, forming a flexible and transparent device. Furthermore, the fabrication process of this hybrid structure is simple, inexpensive, and large-scale because it is a solution-based process.

Herein, we report the fabrication and characterization of a graphene/PbS QD hybrid structure for application in near-infrared PDs. Graphene flakes were dispersed in butylamine and hexane to achieve uniform distribution on a glass substrate. Hexane solution was used to disperse the PbS QDs. Mixtures of graphene flakes and PbS QDs were deposited on a glass substrate using a spin casting machine. Further, the room-temperature photoluminescence, and the Raman and absorption spectra were measured to confirm the optical properties that can be modified by the photoinduced charge transfer of PbS QDs. The surface morphology was evaluated using scanning electron microscopy. The device performance was assessed using the current–voltage curve, photocurrent measurement, and external quantum efficiency.

## Results

**Figure 1:**
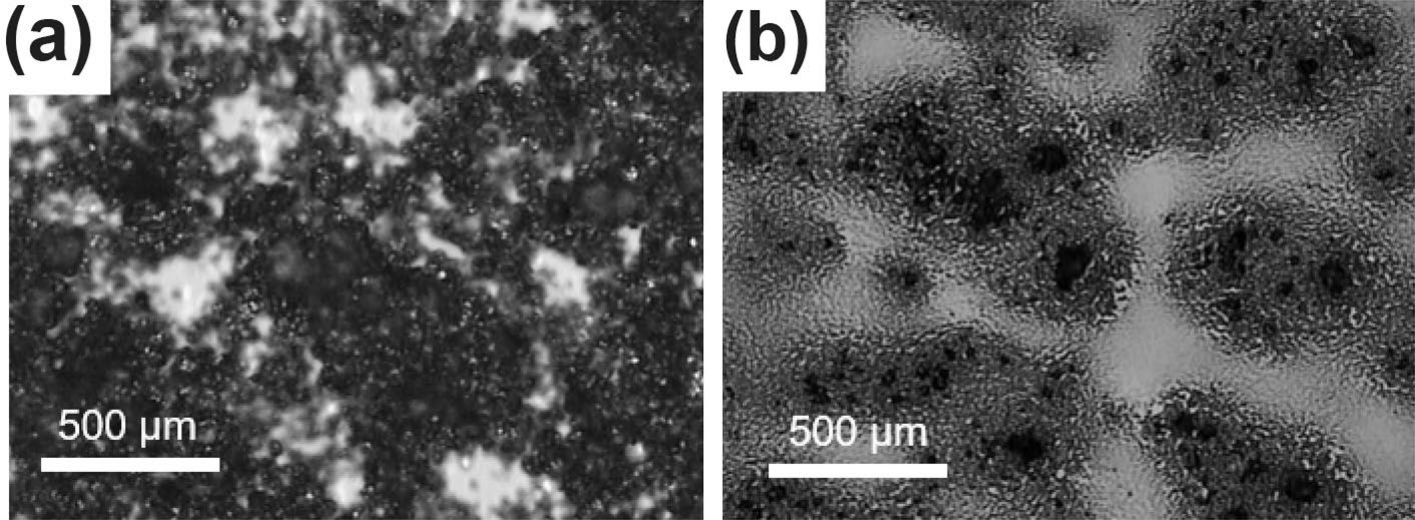
Optical photographs of the graphene-PbS QD composite: Optical microscopy images of the (**a**) graphene flakes and (**b**) graphene-PbS QD composite dispersed on a glass substrate.

In this study, single-layer graphene and PbS QD composite was dispersed in hexane solution. (See Method section) The single-layer graphene and PbS QD composite was coated on a glass substrate treated with ultraviolet-ozone surface cleaning for 10 min. Figure 1a,b are the optical photographs of graphene alone, and the graphene and PbS QD composite coated on a glass substrate, respectively; the scale bar for both images is 500 μm. In the figures, meaningfully different surface images can be observed, establishing that the PbS QDs are physically and/or chemically attached to the graphene flakes.

**Figure 2:**
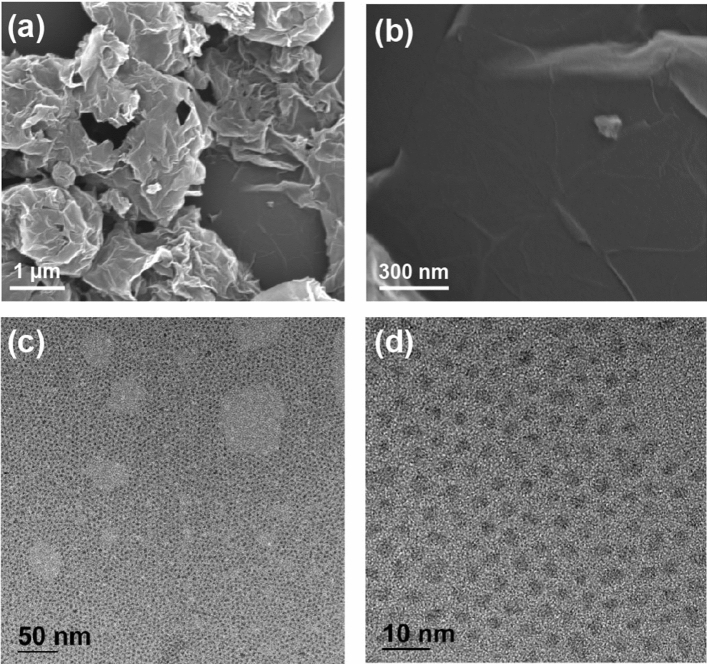
SEM images of the graphene-PbS QD composite and TEM images of the PbS QDs: SEM images of the graphene-PbS QD composite at (**a**) low and (**b**) high magnification. TEM images of the PbS QDs at (**c**) low and (**d**) high magnification.

Figure 2a,b display the field emission scanning electron microscopy (FESEM) images of the graphene-PbS QD composite coated on a glass substrate, at low and high magnification, respectively. The combination of aggregated graphene flakes and PbS QDs, invisible in the SEM images, is randomly distributed on the glass substrate (Fig. 2a). Single-layer graphene flakes are also observed on a partial area of the glass substrate. (Fig. 2b). In addition, the white wrinkles visible in Fig. 2b are a conventional characteristic of single-layer graphene (see Supporting Information Fig. SI1). The PbS QD and graphene composite was investigated in further detail by transmission electron microscopy (TEM). Figure 2c,d display the TEM images of the PbS QDs synthesized in this study, at low and high magnifications, respectively. Closely-packed PbS QDs were clearly observed in the TEM images (Fig. 2(c)) with considerably uniform sizes, indicating that the energy bandgaps of the individual QDs were consistent (Fig. 2d)^28^.

For the quantitative examination of the graphene-PbS QD composite, energy dispersive spectroscopy (EDS) was employed. Table 1 shows element analysis of graphene-PbS QD composite using EDS. While atomic percent of graphene (C) is 48.37 at.%, those of Pb and S elements are 2.36 at.% and 2.16 at.%. Relatively smaller atomic percent of PbS QDs than that of graphene is attributed by smaller volume fraction of PbS QDs in the unit area. Nevertheless, PbS QDs have considerable atomic percent in the graphene-PbS QD composite to be acted as an active material of photo-responsive devices. In addition, elements of Si and O are from glass substrate.

**Table 1:** Elemental analysis of graphene-PbS QDs composite using EDS.

Element	Atomic percent (At%)
C	48.37
Pb	2.36
S	2.16
Si	12.21
O	27.29
Etc	7.61

**Figure 3:**
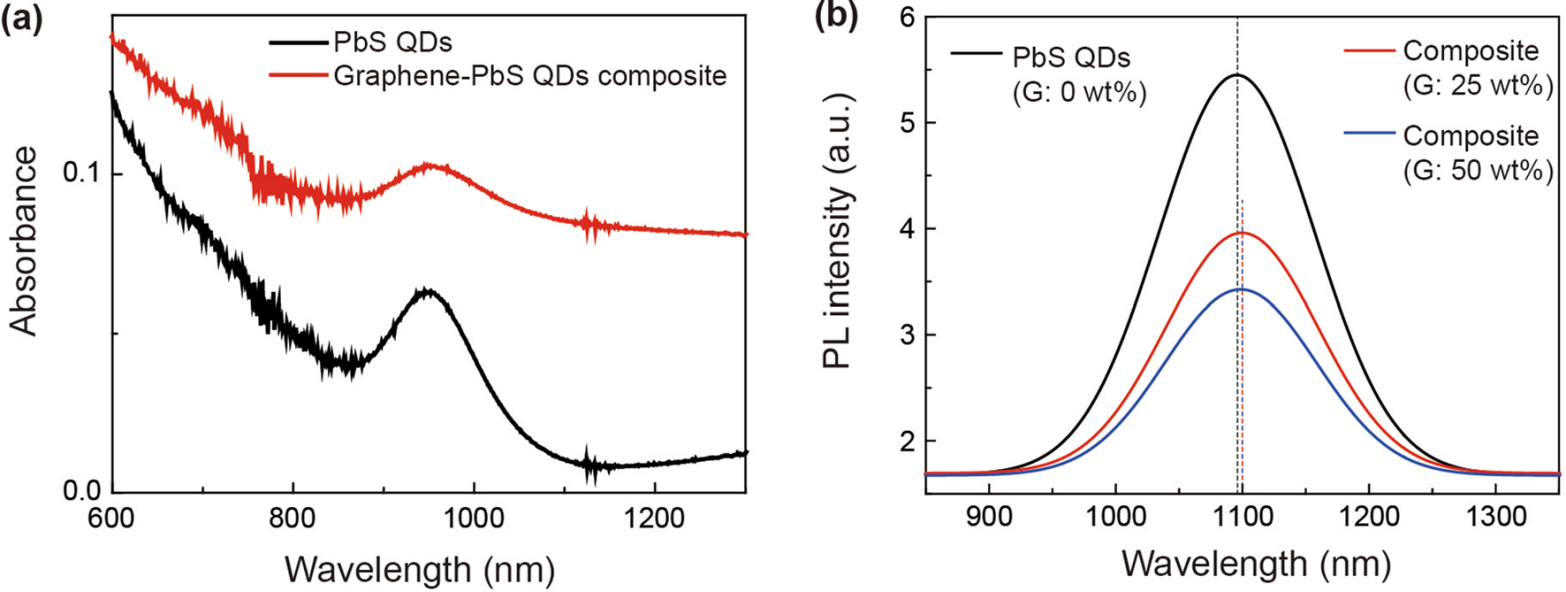
Absorption and photoluminescence (PL) spectra: (**a**) Absorption spectra of the PbS QDs and graphene-PbS QD composite. (**b**) PL spectra of the graphene-PbS QD composite according to the quantitative rate of graphene.

The optical properties of the graphene-PbS QD composite were investigated through absorption and photoluminescence (PL) spectroscopies. Figure 3a presents the absorption spectra of the PbS QDs and the graphene-PbS QD composite, denoted by red and black solid lines, respectively. The peak wavelengths of both samples at 950 nm correspond to the band gap energy of the PbS QDs. However, the absorbance of the graphene-PbS QD composite is greater than that of the Pbs QDs alone because of enhanced photoinduced charge transfer with the active layer^29^. Note that this increase of absorbance is not mainly induced by absorption of multiple layers of graphene because absorbance of graphene/PbS QD composite is smaller than that of PbS QDs alone in the wavelength range of shorter than 565 nm. (see Supporting Information Fig. SI2).

To further investigate the photoinduced charge transfer phenomenon exhibited by the graphene-PbS QDs, PL measurements were performed using a 325 nm-wavelength He-Cd laser as the excitation source. Figure 3b displays the room-temperature PL spectra of PbS QDs combined with 0 wt%, 25 wt%, 50 wt% graphene, denoted by black, red, and blue solid lines, respectively, in the graph. The PL peak wavelengths of the PbS QDs and graphene-PbS QD composite were 1,095 nm and 1,100 nm, respectively. The red-shifted PL peak of the graphene-PbS QD composite is attributed not only to photoinduced charge transfer but also to the spontaneous doping effect of the PbS QDs due to the transferred carriers between them and graphene^30^. The PL intensities of the graphene-PbS QD composites with 25% and 50% graphene were lesser by 24.3% and 37.2%, respectively, compared to that of the PbS QDs. The PL intensity represents the number of recombined carriers in the PbS QDs for a unit amount of QDs. These quenched PL intensities are attributed to the enhanced transfer of the photoinduced carriers between graphene and the PbS QDs, which minimizes the charge recombination rate. In addition, Fourier-transform infrared spectroscopy establishes that hexane solution and the glass substrate do not affect the optical properties of the PbS QDs and graphene flakes. (see Supporting Information Fig. SI3).

**Figure 4:**
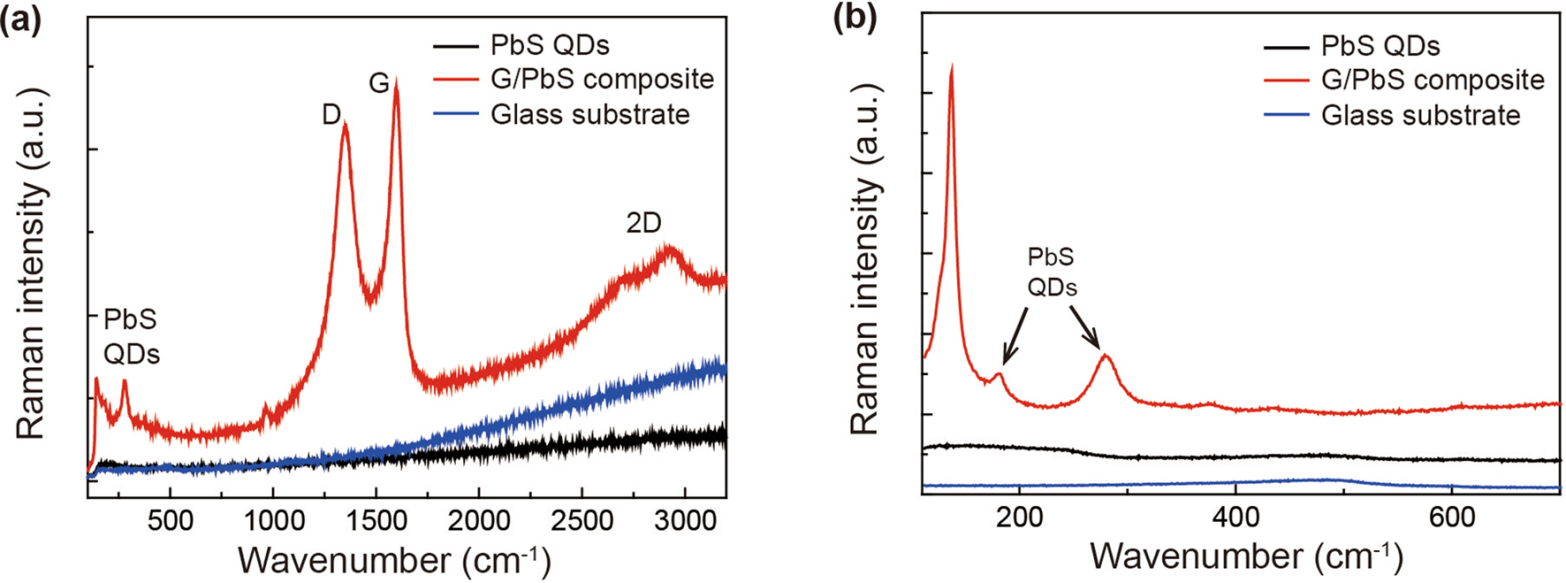
Raman scattering of the PbS QDs, graphene-PbS QD composite, and glass substrate: Micro-Raman spectra measured using (**a**) 150 and (**b**) 3,000 groove gratings.

Micro-Raman scattering analysis was performed to examine the photoinduced charge transfer between graphene and the PbS QDs, with a He–Ne laser operating at a wavelength of 632.8 nm. The Raman spectra of the PbS QDs, graphene-PbS QD composite, and glass substrate depicted in Fig. 4a,b were measured with different reflection-grating groove numbers; the black, red, and blue solid lines denote the Raman spectra of the PbS QDs, graphene-PbS QD composite, and glass substrate, respectively. Figure 4a exhibits the Raman spectra of samples with wavenumbers ranging between 150–3,150 cm^−1^ (150-groove grating). For graphene, three intensive Raman modes are observed at 1,344.8, 1601.3, and 2,701.8 cm^−1^ , corresponding to the D-band, G-band, and 2D-band, respectively^31^. In the Raman spectrum of the graphene-PbS QD composite, two Raman modes are observed at 140.3 and 280.8 cm^−1^, which are associated with the PbS QDs, whereas the Raman spectrum of the PbS QDs does not exhibit a Raman mode^32^. This is because the specific Raman mode embedded in the spectrum of the PbS QDs is enhanced, which is relevant to the photoinduced charge transfer between the Pbs QDs and graphene, as interpreted by SERS. To verify the enhanced Raman mode of the PbS QDs with graphene, micro-Raman spectroscopy was performed using a 3,000-groove grating for high-spectral resolution, as shown in Fig. 4b. The Raman mode observed at 280.0 cm^−1^ is a longitudinal optical (LO) phonon mode corresponding to the 1LO phonon mode of the PbS QD^33,34^. The Raman mode at 181.7 cm^−1^ is attributed to a surface phonon, whose intensity varies depending upon the QD size^35^. The peak at 140.3 cm^−1^ is not relevant to the PbS QD. Thus, it can be deduced that the Raman mode of PbS QDs in combination with graphene is enhanced by the photoinduced charge transfer between the two materials.

**Figure 5:**
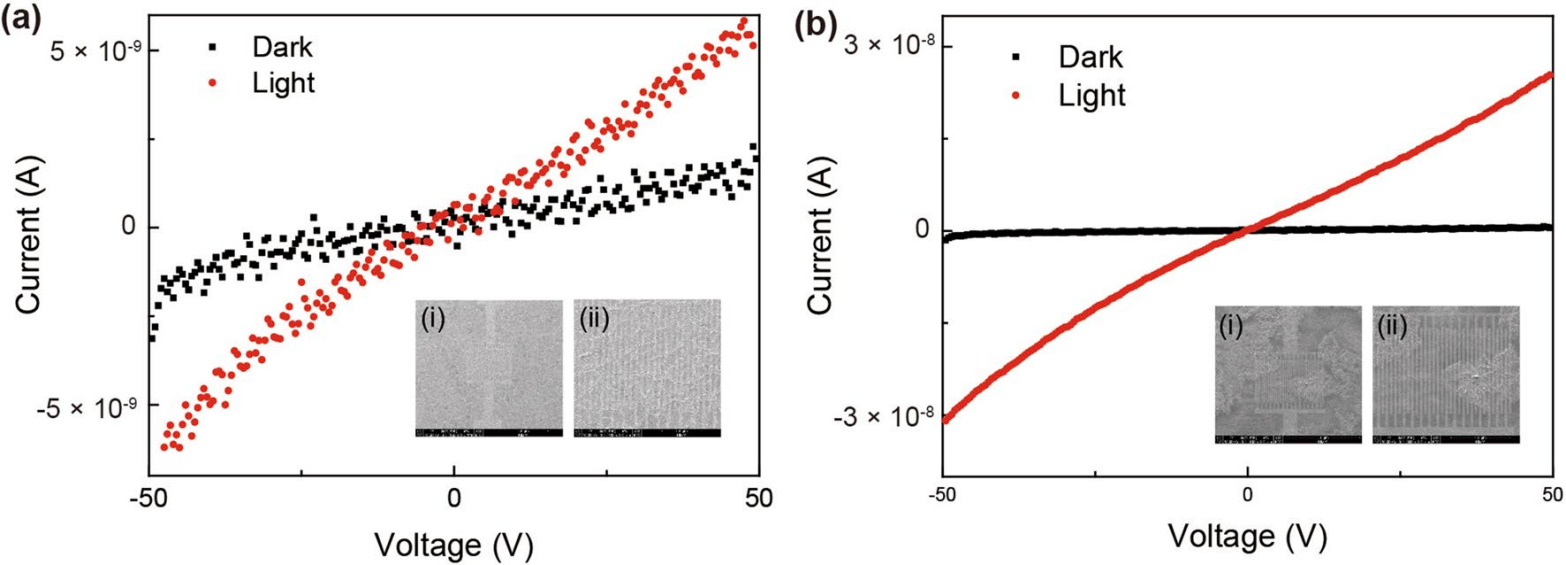
I–V characteristics of photodiodes comprising graphene-PbS QD composite and PbS QDs alone. I–V curves of photodiodes comprising (**a**) PbS QDs alone and (**b**) graphene-PbS QD composite, with and without light illumination on the device surface.

To estimate the photocurrents of PDs comprising PbS QDs and graphene-PbS QD composite, respectively, I–V measurements were performed. For device fabrication, interdigited finger patterns were created by photolithography on an SiO_2_ substrate. For a robust metal pad, Au was deposited on the patterned sample using a thermal evaporator (see Supporting Information Fig. SI3). A 980-nm-laser diode with an optical power of 25 mW was used as the light source and a beam with diameter of 5 mm was illuminated on the sample. Voltages ranging between -50–(+ 50) were applied to all the samples. Figure 5a displays the I–V curves of the PD comprising PbS QDs filled between the interdigited finger electrodes, with and without light illumination. Insets (i) and (ii) show the SEM images of the PD comprising PbS QDs at low and high magnification, respectively; the red and black dotted lines denote the I–V curves of the PD comprising PbS QDs, with and without light illumination, respectively. The currents at 50 V with and without light illumination were 5.4 nA and 1.9 nA, respectively, indicating that a photocurrent of 3.5 nA was generated at an applied voltage of 50 V. The I–V curves of a PD comprising graphene-PbS QDs with and without light illumination, denoted by red and black dotted lines, respectively, are presented in Fig. 5b. For the I–V curve measured with light illumination, the current at − 50 V was 30.9 nA, while that at + 50 V was 25.7, which are approximately five times greater than those of a PD comprising PbS QDs alone. Moreover, the current measured without light illumination at 50 V was 0.5 nA, which is comparable with that of a PD comprising PbS QDs only. This indicates that the photocurrent generation of PbS QDs is enhanced by the combination with graphene. The SEM images of the device surface at low and high magnification are shown in insets (i) and (ii), respectively, in which the assembled graphene flakes attached to the PbS QDs can be clearly observed.

**Figure 6:**
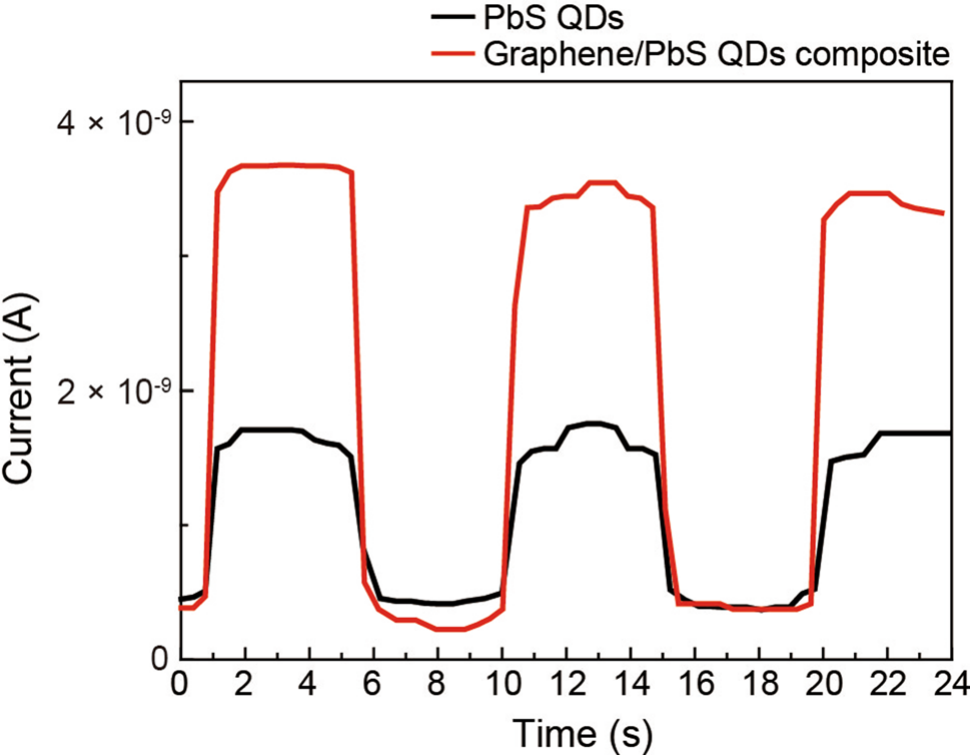
Transient photocurrent response: Time-resolved photoresponses of PbS QDs alone and graphene-PbS QD composite.

To evaluate the device performance, the repeatability and response speed of PDs comprising graphene-PbS QD composite and PbS QDs alone were studied. The PD transient photocurrent response to on/off illumination cycles was measured under an optical power density of 31.8 mW/cm^−1^ with a fixed bias of 10 V. Figure 6 exhibits the time-resolved photoresponse of PDs comprising graphene-PbS QD composite (red circle line) and PbS QDs (black square line). For both PDs, reproducible and stable photocurrents were obtained by repeated on–off light illumination cycles, and the current measured without illumination was 0.4 nA. The current of the PD comprising graphene-PbS QD composite, with light illumination, was 3.6 nA on an average. On the other hand, the current of the PD comprising PbS QDs alone was 1.7 nA, which is approximately lesser by twice compared to that of the PD comprising graphene-PbS QD composite. The rise and decay times of the photocurrent were extracted from the transient photocurrent response by fitting with an exponential function. The rise time and decay time of the PD comprising PbS QDs are 214 and 350 ms, respectively. For the PD comprising graphene-PbS QD composite, the rise and decay time are 167 and 187 ms, respectively, which are significantly shorter than those of the PD comprising Pbs QDs alone. This faster response time of the PD comprising graphene-PbS QD composite is attributed to the photoinduced charge transfer between graphene and the PbS QDs.

In conclusion, we presented a graphene-PbS QD composite for application in near-infrared PDs. Single-layer graphene flakes and oleic acid-capped PbS QDs were combined using a conventional sonication process, in hexane solution. FESEM images of the graphene-PbS QDs composite coated on a glass substrate confirm that the composite contains both aggregated graphene flakes and single-layer graphene with wrinkles; TEM images confirm close packing with uniform size. The increased absorbance and quenched PL intensity of the graphene-PbS QD composite supports enhanced photoinduced charge transfer between graphene and the PbS QDs. Moreover, in the spectrum, the specific Raman mode of the PbS QDs was enhanced by combination with graphene; this is relevant to the photoinduced charge transfer between the Pbs QDs and graphene, as interpreted by SERS. For device application, a PD structure comprising graphene-PbS QDs was fabricated. The photocurrent of the PD was measured using a conventional probe station with a 980-nm near-infrared (NIR) laser diode. In the PD comprising graphene-PbS QD composite, five-times higher photocurrent, 22% shorter rise time, and 47% shorter decay time were observed, compared to that comprising PbS QDs alone. This establishes that the graphene-PbS QD composite has significant potential for application in ultrathin, flexible, high-performance NIR PDs.

## Methods

### Preparation of graphene flakes

Single-layer graphene flakes (ACS Material) are fabricated using the modified Hummer’s method. The average diameter and thickness of these graphene flakes ranged between 0.4–0.5 μm and 0.6–1.2 nm, respectively. Single-layer graphene flakes were dispersed in hexane solution (2 mg/ml) through a sonication process for 10 min.

### Synthesis of PbS QDs

All the manipulations for preparing the PbS QDs were performed using the standard Schlenk line technique. Oleic acid-capped PbS QDs (first exciton peak at 950 nm) were prepared as per a previously reported method^36,37^. Subsequently, post-treatment and purification were performed following a published method with some modifications^38^:

### Device fabrication

Interdigital electrodes were fabricated on an SiO_2_ substrate through conventional photolithography and thermal evaporation. A 300-nm thick Au layer was employed as the electrode. Ligand exchanges of the PbS QDs were realized using 1,2-ethanedithiol (EDT) to improve device carrier transportation.

### Characterization

The absorption spectrum was obtained using a UV–Visible/NIR spectrophotometer (JASCO). PL spectroscopy was performed using a 30-cm monochromator and a charge-coupled device detector excited by a 355-nm diode-pumped solid-state laser. The micro-Raman spectrum was measured with a backscattering geometry using a He–Ne laser (633 nm) for excitation.

## Supplementary information

Supplementary Information.
