# Supplementary material for: Graphene/PbS quantum dot hybrid structure for application in near-infrared photodetectors
Source: Sci Rep. 2020 Jul 27;10:12475. doi: 10.1038/s41598-020-69302-6 (PMC7385648; doi:10.1038/s41598-020-69302-6)
Supplement: Supplementary file 1 — Supplementary Information. [file 41598_2020_69302_MOESM1_ESM.doc]

Supporting Information

Graphene/PbS quantum dot hybrid structure for application in near-infrared photodetectors

Hyun Jeong^1^, Jung Hoon Song,^2^ Sohee Jeong,^2^ and Won Seok Chang^1,3*^

*Correspondence to [paul@kimm.re.kr]

^1^Department of Nanoprocess, Korea Institute of Machinery and Materials (KIMM), Daejeon 34103, Republic of Korea, ^2^Department of Energy Science (DOES), Sungkyunkwan University (SKKU), Suwon 16419, Republic of Korea, ^3^Department of Nano-Mechatronics, Korea University of Science and Technology (UST), Daejeon 34113, Republic of Korea

**SI1. Scanning electron miscroscopy (SEM) and energy dispersive spectroscopy (EDS)**

1.1. SEM image and EDS spectrum of the graphene flakes

**
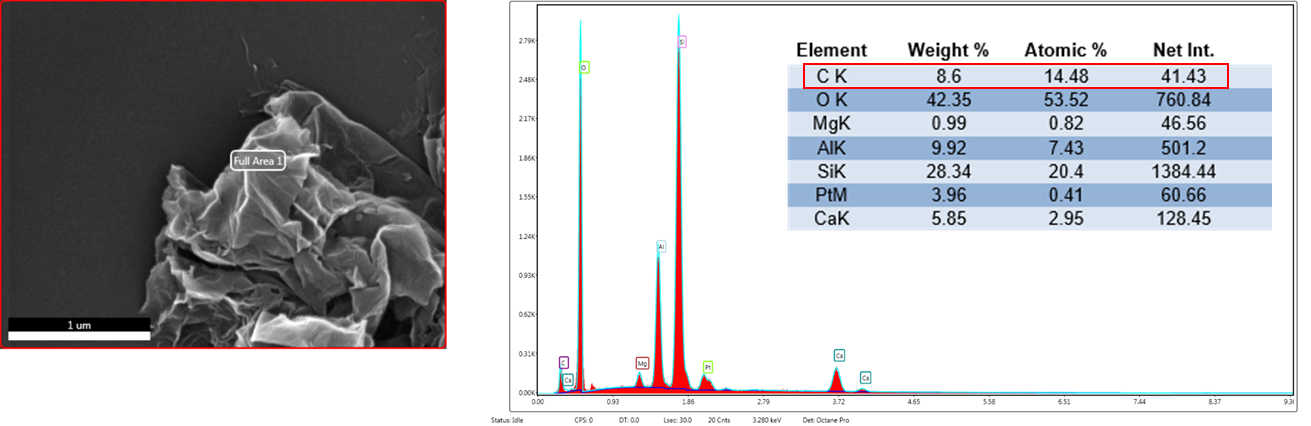
**

1.2. SEM image and EDS spectrum of the graphene-PbS QDs composite

**
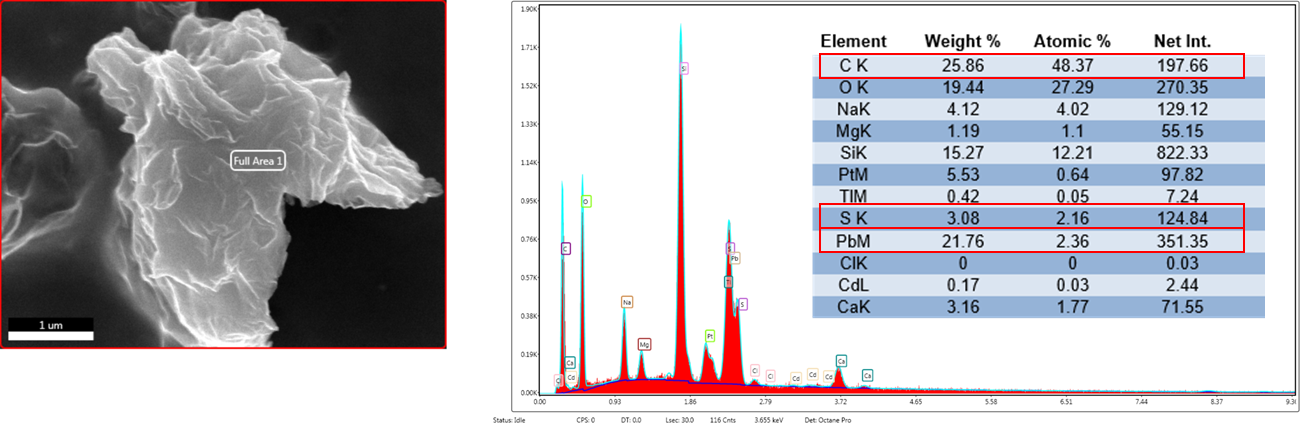
**

**SI2. Absorption spectra** **the PbS QDs and graphene-PbS QDs composite.**

**
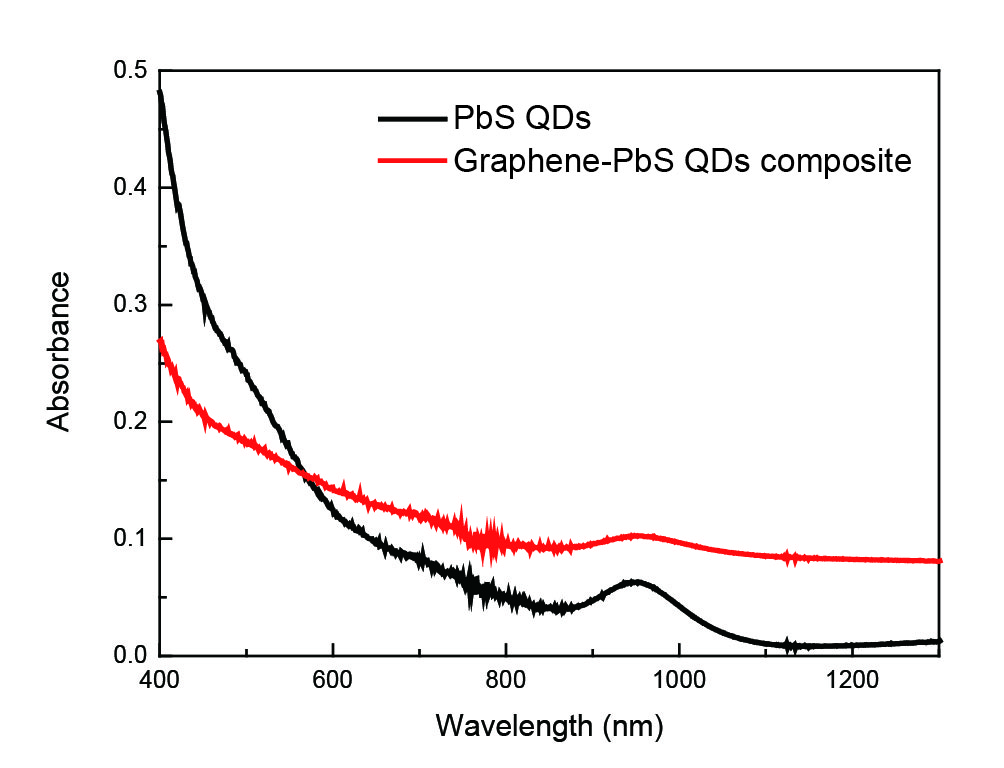
**

**SI3.** **Fourier-transform infrared spectroscopy**

**
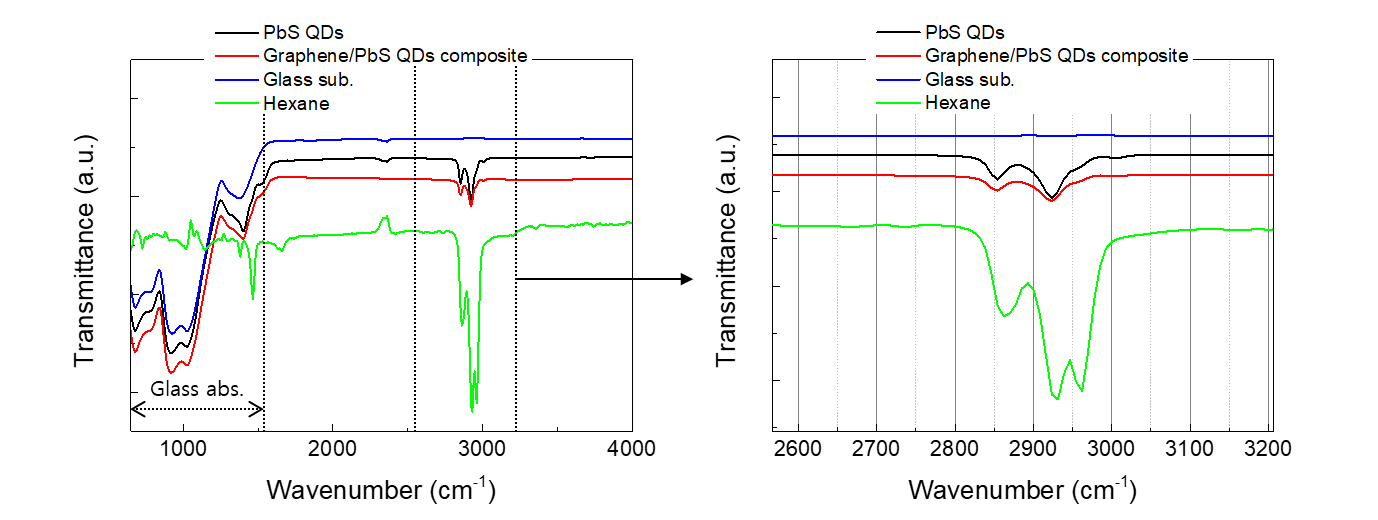
**

**SI3. SEM image of the device structure (QDs alone, Graphene+QDs)**

3.1. Interdigital electrode structure covered by PbS QDs alone

**
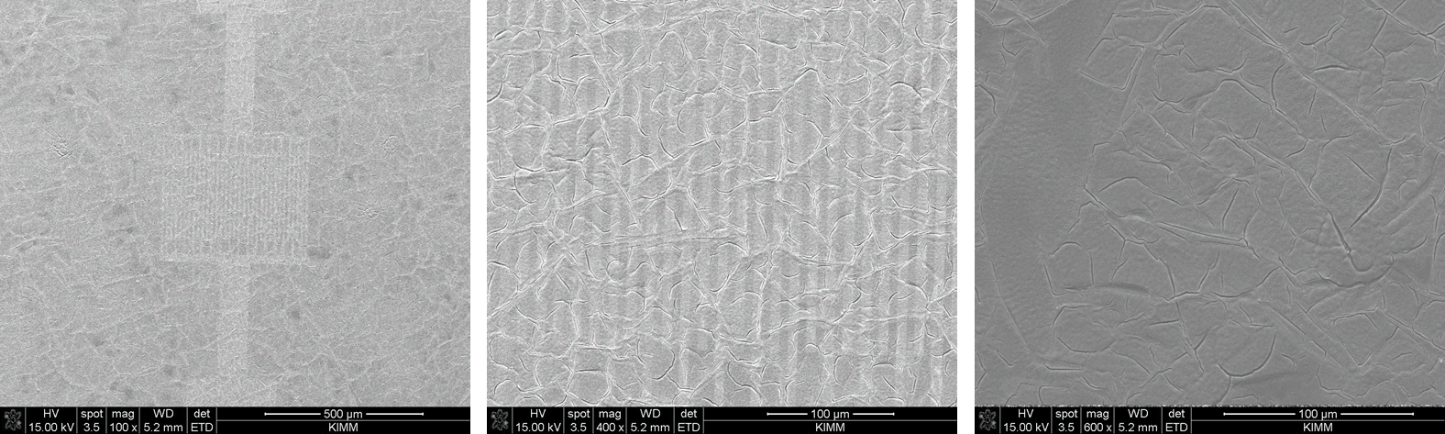
**

3.2. Interdigital electrode structure covered by the graphene-PbS QD composite

**
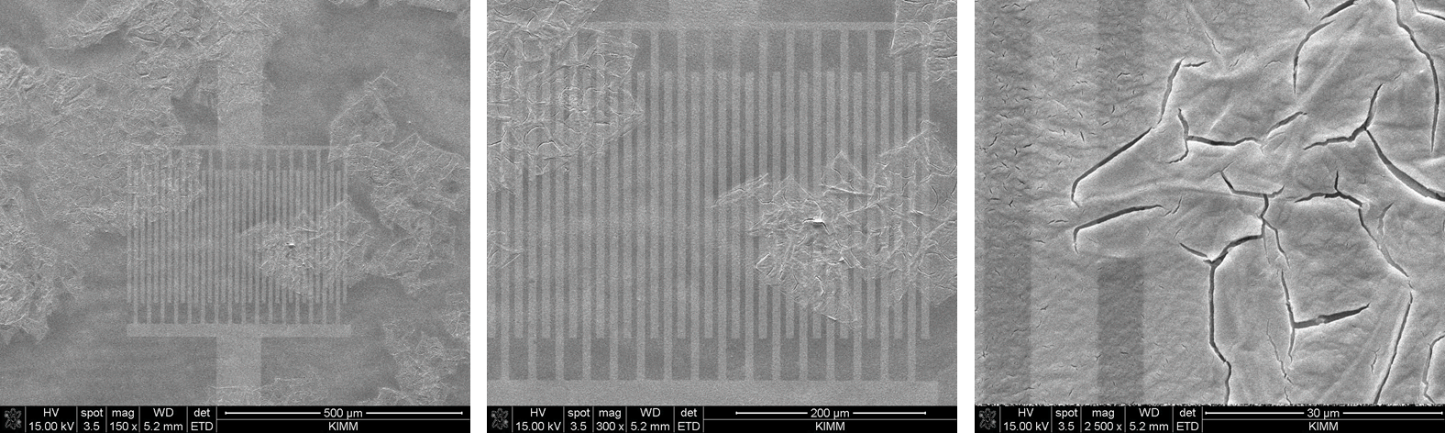
**
